# Meta-analysis on the effectiveness of team-based learning on medical education in China

**DOI:** 10.1186/s12909-018-1179-1

**Published:** 2018-04-10

**Authors:** Minjian Chen, Chunhui Ni, Yanhui Hu, Meilin Wang, Lu Liu, Xiaoming Ji, Haiyan Chu, Wei Wu, Chuncheng Lu, Shouyu Wang, Shoulin Wang, Liping Zhao, Zhong Li, Huijuan Zhu, Jianming Wang, Yankai Xia, Xinru Wang

**Affiliations:** 1 Department of Occupational Medicine and Environmental Health, School of Public Health, Nanjing Medical University, No. 101 Longmian Road, Nanjing, 211166 China; 2 Experimental Teaching Center of Preventive Medicine, Nanjing Medical University, Nanjing, 211166 China; 3 Safety Assessment and Research Center for Drug, Pesticide and Veterinary Drug of Jiangsu Province, Nanjing Medical University, Nanjing, 211166 China; 4 Department of Student Affairs, School of Public Health, Nanjing Medical University, Nanjing, 211166 China; 5 Department of Toxicology, School of Public Health, Nanjing Medical University, Nanjing, 211166 China; 6 Office of School of Public Health, Nanjing Medical University, Nanjing, 211166 China; 7 Department of Nutrition and Food Hygiene, School of Public Health, Nanjing Medical University, Nanjing, 211166 China; 8 Department of Epidemiology, School of Public Health, Nanjing Medical University, Nanjing, 211166 China

**Keywords:** TBL, LBL, Medical education, China, Meta-analysis

## Abstract

**Background:**

Team-based learning (TBL) has been adopted as a new medical pedagogical approach in China. However, there are no studies or reviews summarizing the effectiveness of TBL on medical education. This study aims to obtain an overall estimation of the effectiveness of TBL on outcomes of theoretical teaching of medical education in China.

**Methods:**

We retrieved the studies from inception through December, 2015. Chinese National Knowledge Infrastructure, Chinese Biomedical Literature Database, Chinese Wanfang Database, Chinese Scientific Journal Database, PubMed, EMBASE and Cochrane Database were searched. The quality of included studies was assessed by the Newcastle-Ottawa scale. Standardized mean difference (SMD) was applied for the estimation of the pooled effects. Heterogeneity assumption was detected by *I*^2^ statistics, and was further explored by meta-regression analysis.

**Results:**

A total of 13 articles including 1545 participants eventually entered into the meta-analysis. The quality scores of these studies ranged from 6 to 10. Altogether, TBL significantly increased students’ theoretical examination scores when compared with lecture-based learning (LBL) (SMD = 2.46, 95% CI: 1.53–3.40). Additionally, TBL significantly increased students’ learning attitude (SMD = 3.23, 95% CI: 2.27–4.20), and learning skill (SMD = 2.70, 95% CI: 1.33–4.07). The meta-regression results showed that randomization, education classification and gender diversity were the factors that caused heterogeneity.

**Conclusions:**

TBL in theoretical teaching of medical education seems to be more effective than LBL in improving the knowledge, attitude and skill of students in China, providing evidence for the implement of TBL in medical education in China. The medical schools should implement TBL with the consideration on the practical teaching situations such as students’ education level.

**Electronic supplementary material:**

The online version of this article (10.1186/s12909-018-1179-1) contains supplementary material, which is available to authorized users.

## Background

To improve the education effectiveness, the integrated implementing of traditional lecture-based learning (LBL) pedagogy and new pedagogical approaches as supplementary teaching methods has been a trend in Chinese medical teaching in recent years. Therefore, there is a great need to explore new pedagogical approach which could be introduced in medical education in China based on scientifically validation of its teaching effectiveness.

Team-based learning (TBL), a pedagogical model of small-group learning, was originally developed by Dr. Larry Michaelsen for use in business schools [1]. TBL was an increasingly popular style of active learning pedagogical approach around the world [2]. In traditional LBL pedagogy, students mainly memorize the content from the class lecturer, while TBL is a pedagogical method of active learning. TBL enhances students’ learning motivation, and then impels students to apply these knowledge materials to solve problem and combine theory with practice [2, 3]. At present, multiple medical schools have adopted TBL pedagogical approach globally [4, 5]. For example, some medical schools in Japan, Korea, India, Singapore, Oman, the USA, Lebanon and Australia already have adopted TBL pedagogical approach [6–12].

In China, the most commonly used teaching method in medical education is still LBL. In the past decade, the health and medical education system in China has been developing rapidly. The introduction of active learning pedagogical approach into medical education attracts attention. TBL has many significant advantages fitting for the status of Chinese medical education. TBL is an active learning pedagogical approach, and it permits a large student-teacher ratio, which greatly fits for the status of Chinese medical education with the lack of teachers and classrooms [13]. In recent years, TBL as an emerging pedagogical approach has also been introduced in some medical schools in China. There are also studies regarding the teaching effectiveness of TBL in medical education. Notably, in China, besides exercitation period, the medical teaching is divided into theory course and laboratory course, which are helpful for the improvement of students theoretical knowledge and practical ability, respectively. Theoretical teaching is the basis for practice. In medical education in China, theory course often occupies the most teaching time with the largest proportion of learning contents in the final examination. The only method is often LBL in theory course in China, while the current teaching method in laboratory course is more complex. Students are often divided into groups to practice and do experiment together in the laboratory course. In addition, the theoretical examination is often conducted by an standardized written test which is objective, while some practical examinations for laboratory course still need to be standardized to avoid subjectivity. Given the significance and educational situation in China, in the published studies, the comparison on teaching effectiveness was mostly conducted between TBL and LBL based on theoretical examination. The above information indicates the opportunity and the importance to compare the teaching effectiveness between TBL and LBL in theoretical teaching of medical education.

However, there are still differences in the findings about teaching effectiveness of TBL in the published studies, and the sample size in these studies was relatively small. A pooled analysis of these studies using meta-analysis can solve above problems, and can provide new insights into the implementation of TBL and an important scientific basis for improving medical education in China. However, until now, there are no studies or reviews summarizing the effectiveness of TBL on medical education in China by meta-analysis.

In present study, a summary analysis of 13 studies was conducted to obtain an overall estimation of the effectiveness of TBL on outcomes of theoretical teaching of medical education in China. Because the paper on TBL in medical education in China was mostly published in Chinese which cannot be accessed by non-Chinese-speaking researchers, this meta-analysis can also disseminate TBL implement significance in medical education in China to international education researchers. Moreover, the findings of the meta-analysis in China can provide the first-hand overall understanding of the teaching effectiveness of TBL in medical education, which may also be referable for other countries especially those countries having similar pedagogical structures as China.

## Methods

### Study design

In this study, we planned and conducted meta-analysis following the guidelines of preferred reporting items for systematic review and meta-analysis protocols 2015 statement recommendations [14].

### Literature search

We retrieved the studies from inception through December, 2015. Chinese National Knowledge Infrastructure (CNKI), Chinese Biomedical Literature Database (CBM), Chinese Wanfang Database, Chinese Scientific Journal Database (VIP) were searched. English databases including PubMed, EMBASE and Cochrane Database were also searched. The following key words were used: team-based learning, TBL, theory, theoretical, China, Chinese, medicine, medical, disease, health, healthy, biology, biological, hygiene, hygienic, pharmacology, pharmacological. Additionally, all articles were included by manual operation, and studies matching the eligible criteria were retrieved for further data extraction and quality assessment.

### Inclusion criteria

Eligible studies were required to meet the following explicit inclusion criteria: 1) TBL pedagogy courses should be medicine professional disciplines; 2) the study should be designed as a randomized or nonrandomized trial; 3) the study should compare the effectiveness of TBL and LBL methods by theoretical examination based on the centesimal system; 4) the study had detailed quantitative results for TBL group and LBL group, and data should be available. In this study, data for meta-analysis were available from 13 studies, including 1545 participants.

### Data extraction and quality assessment

According to the inclusion criteria listed above, two investigators independently extracted the data. The two investigators compared their results of data extraction to determine whether there was a disagreement. In cases of disagreement, the third investigator reviewed the study, and a consensus was reached by conference among the 3 investigators. The following information was extracted from all eligible studies: first author’s name, year of publication, the discipline, total number of participants in TBL group and LBL group, source of participants, and the outcome assessment.

Many nonrandomized trials were included. Therefore, the quality of included studies was assessed by the Newcastle-Ottawa scale in the meta-analysis, which was judged in the following items: participant number (1–3), randomization (0–1), blinding (0–1), allocation concealment (0–1), control for important factors (0–2), control for incomplete data bias (0–1), and outcome assessment (0–2). The full score was 11 points, while study of 5 or more points was judged as a high-quality study.

### Statistical pooling and evaluation of heterogeneity

For continuous data, standardized mean difference (SMD) was applied for the estimation of the pooled effects on learning outcomes. Heterogeneity assumption was detected by *I*^2^ statistics. While a *P*-value ≤0.10 in the *I*^2^ test, the random-effects model (DerSimonian and Laird method) was employed [15]. Otherwise, we adopted the fixed-effects model (the Mantel-Haenszel method) [16]. The heterogeneity was further explored by meta-regression analysis based on restricted maximum likelihood [17]. Quality scores and student numbers were modeled as continuous variables; randomization, allocation concealment, control for important factors, source of participants (medical college or undergraduate students), discipline (or curricula) and gender were treated as categorical variables. Subgroup analysis was conducted based on the results of meta-regression analysis.

### Sensitivity analyses

In order to evaluate the statistical robustness of the results, a single study in the meta-analysis was deleted each time to show the effect of the individual data set on the pooled results, and we also used fixed-effects model to assess the stability of the results.

### Publication bias evaluation

Publication bias was assessed using a funnel plot and Begg’s test according to previous reports [18]. All analyses were carried out with Stata statistical software (version 11.0, StataCorp LP-College Station, TX, USA).

## Results

### Study characteristics and quality assessment

**Fig. 1:**
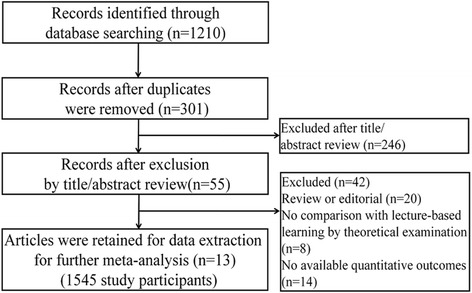
Flow chart for the inclusion of studies for meta-analysis

The inclusion process of all studies is shown in Fig. 1. A total of 1210 records in Chinese or English were retrieved based on the search strategy, and 909 duplicate records were next removed. After reviewing the title/abstract, 55 articles were retained for further examination. According to the inclusion criteria, 42 articles were excluded (20 were reviews or editorials; 8 included no comparison with LBL; 14 had no available quantitative outcomes). A total of 13 articles including 1545 participants eventually entered into the meta-analysis [19–31], and they were all written in Chinese. Table 1 shows the study characteristics of the 13 studies. Most of the studies (12/13) reported the admission years of the students ranging from 2008 to 2011. Only 2 out of the 13 studies reported the study years in 2010 and 2013 for Xu et al. [29] and Tao et al. [26], respectively.

**Table 1:** Characteristics of included studies

First author (Publication year)	Disciplines or curricula	TBL participants	LBL participants	Total number	Gender	Source of participants (Major)	Outcome assessment
Wu et al. (2015) [19]	Periodontics	32	32	64	Male+Female	Undergraduate students (Stomatology)	Examination scores and questionnaire surveys
Yuan (2014) [20]	Medical English	96	98	194	Male+Female	Undergraduate students (Clinical medicine)	Examination scores and questionnaire surveys
Zhu (2014) [21]	Regional anatomy	90	90	180	Male+Female	Undergraduate students (Clinical medicine)	Examination scores and questionnaire surveys
Chao et al. (2013) [22]	Preventive medicine	39	41	80	Male+Female	Undergraduate students (Nursing)	Examination scores and questionnaire surveys
Huang et al. (2013) [23]	Medical English	48	49	97	Female	Medical college students (Nursing)	Examination scores and questionnaire surveys
Liu & Zhang (2013) [24]	Gynecology and obstetrics	36	33	69	Female	Undergraduate students (Clinical medicine)	Examination scores and questionnaire surveys
Mi et al. (2013) [25]	Nutrition science and food hygiene	40	41	81	Male+Female	Undergraduate students (Preventive medicine)	Examination scores and questionnaire surveys
Tao et al. (2013) [26]	Pathology	56	57	113	Male+Female	Medical college students (Nursing)	Examination scores and questionnaire surveys
Huang et al. (2012) [27]	Histoembryology	40	41	81	Male+Female	Medical college students (Clinical medicine)	Examination scores and questionnaire surveys
Li (2012) [28]	Medical microbiology	135	135	270	Male+Female	Undergraduate students (Clinical medicine)	Examination scores and questionnaire surveys
Xu et al. (2012) [29]	Emergency and critical care nursing	52	50	102	Female	Medical college students (Nursing)	Examination scores and questionnaire surveys
Zhang et al. (2012) [30]	Obstetrics	48	48	96	Female	Medical college students (Nursing)	Examination scores and questionnaire surveys
Wan (2011) [31]	Pathology	60	58	118	Male+Female	Medical college students (Nursing)	Examination scores and questionnaire surveys

*TBL* team based-learning, *LBL* lecture-based learning; Undergraduate students (5-year program); Medical college students (3-year program)

Table 2 shows the methodological quality of the 13 included studies. All these articles were published in peer-reviewed journals. The quality scores ranged from 6 to 10. Bias protection approaches including allocation concealment, control for important factors as well as control for incomplete data bias were often applied in these studies. However, whether the outcome assessors and data collectors were blinded to subjects’ assignments was not mentioned in these studies, and only two studies applied randomization. All studies measured the outcomes by both theoretical examination scores and questionnaire surveys.

**Table 2:** Methodological quality of studies included in the meta-analysis

First author (Publication year)	Student numbers	Randomization	Blind	Allocation concealment	Control for important factors	Control for incomplete data bias	Assessment of outcome	Total quality scores
Wu et al. (2015) [19]	1	1	0	1	2	1	2	8
Yuan (2014) [20]	3	0	0	1	2	1	2	9
Zhu (2014) [21]	3	0	0	1	2	1	2	9
Chao et al. (2013) [22]	1	0	0	1	2	1	2	7
Huang et al. (2013) [23]	2	0	0	1	2	1	2	8
Liu & Zhang (2013) [24]	1	0	0	1	1	1	2	6
Mi et al. (2013) [25]	1	0	0	1	2	1	2	7
Tao et al. (2013) [26]	2	0	0	1	2	1	2	8
Huang et al. (2012) [27]	1	0	0	1	1	1	2	6
Li (2012) [28]	3	1	0	1	2	1	2	10
Xu et al. (2012) [29]	2	0	0	0	2	1	2	7
Zhang et al. (2012) [30]	2	0	0	1	2	1	2	8
Wan (2011) [31]	3	0	0	1	2	1	2	9

Student numbers, a maximum of 3 scores could be awarded for this item. Studies in which student numbers were 50 to 81, 96 to 113, 118 to 270 received 1 score, 2 scores, 3 scores, respectively; Control for important factors, a maximum of 2 scores could be awarded for this item. Studies which were controlled for age received 1 score, and studies which were controlled for previous academic performance received an additional score;

Assessment of outcome, a maximum of 2 scores could be awarded for this item. Studies which were measured by examination scores received 1 score, and studies which were measured by both examination scores and questionnaire surveys received two scores

### Data synthesis

**Fig. 2:**
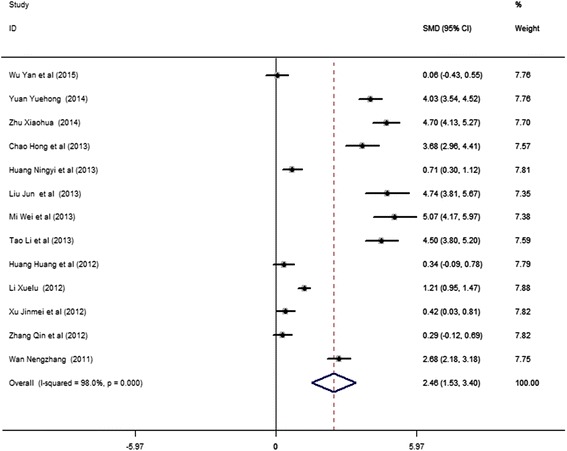
Forest plot for the effect of TBL on theoretical examination scores compared with LBL. Studies are plotted according to the last name of the first author and followed by the publication year in parentheses. Horizontal lines represent 95% CI. Each square represents the SMD point estimate, and its size is proportional to the weight of the study. The diamond (and broken line) represents the overall summary estimate, with confidence interval given by its width. The unbroken vertical line is at the null value (SMD = 0). CI, confidence interval; SMD, standardized mean difference

The effectiveness of TBL on medical education was assessed by pooling theoretical examination scores, and the results on the learning attitude and self-directed learning skill are shown in the present study (Fig. 2, Table 3).

**Table 3:** Summary of effect sizes for TBL and LBL

Outcomes	No. of studies	No. of subjects	SMD (95% CI)	*I* ^2^	*P*_Heterogeneity_
Examination scores	13	1545	2.46 (1.53–3.40)	98.00%	*P* < 0.001
Questionnaire surveys					
Learning attitude	4	505	3.23 (2.27–4.20)	92.10%	*P* < 0.001
Learning skill	5	607	2.70 (1.33–4.07)	97.40%	*P* < 0.001

*TBL* team-based learning, *LBL* lecture-based learning, *SMD* standardized mean difference, *CI* confidence interval;

No. of studies, learning attitude (Yuan et al. [20], Chao et al. [22], Tao [26], Wan [31]); learning skill (Yuan et al. [20], Chao et al. [22], Tao [26], Xu et al. [29], Wan [31]);

SMD (95% CI), random-effects model was used because *P* value for heterogeneity test< 0.10

As the effectiveness of TBL on theoretical examination scores was reported in all the 13 studies, the data on the examination scores of the 13 studies were pooled into the meta-analysis. We found that TBL significantly increased students’ examination scores when compared with LBL in random-effects model (SMD = 2.46, 95% CI: 1.53–3.40, *I*^2^ = 98.0%, *P*_heterogeneity_ < 0.001) (Fig. 2, Table 3). This pooled analysis with heterogeneity was similar to previous published meta-analysis [32]. Begg’s test did not reveal funnel plot asymmetry (*P* = 0.059), making publication bias unlikely [18]. Four and five studies reported the learning attitude and learning skill, respectively. The pooled TBL effects on learning attitude and learning skill were significant in random-effects models (for learning attitude, SMD = 3.23, 95% CI: 2.27–4.20, *I*^2^ = 92.1%, *P*_heterogeneity_ < 0.001; for learning skill, SMD = 2.70, 95% CI: 1.33–4.07, *I*^2^ = 97.4%, *P*_heterogeneity_ < 0.001) (Table 3).

### Test of heterogeneity

**Fig. 3:**
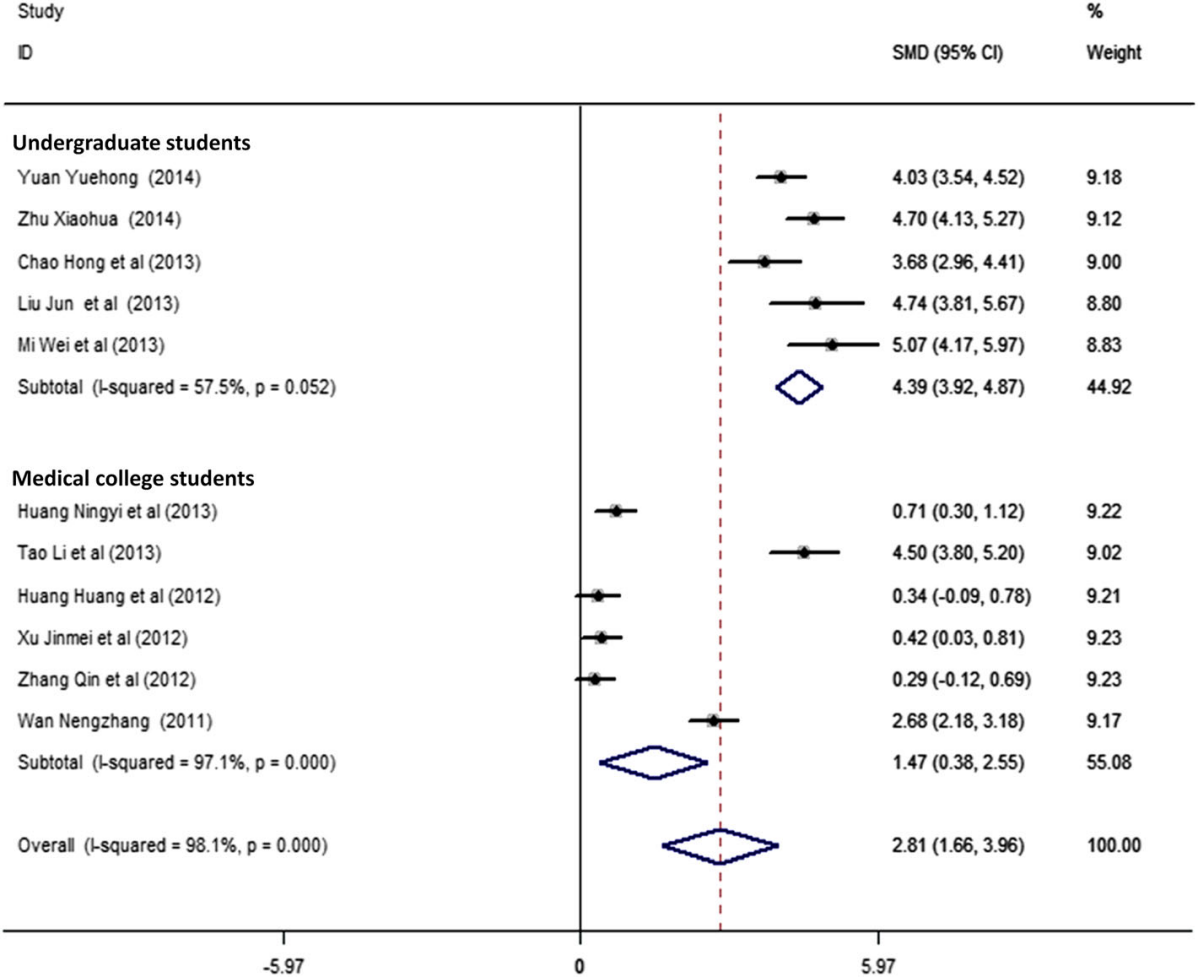
Forest plot for the effect of TBL on theoretical examination scores in nonrandomized studies grouped with education classification. The explanation for forest plot can be found in Fig. 2

**Fig. 4:**
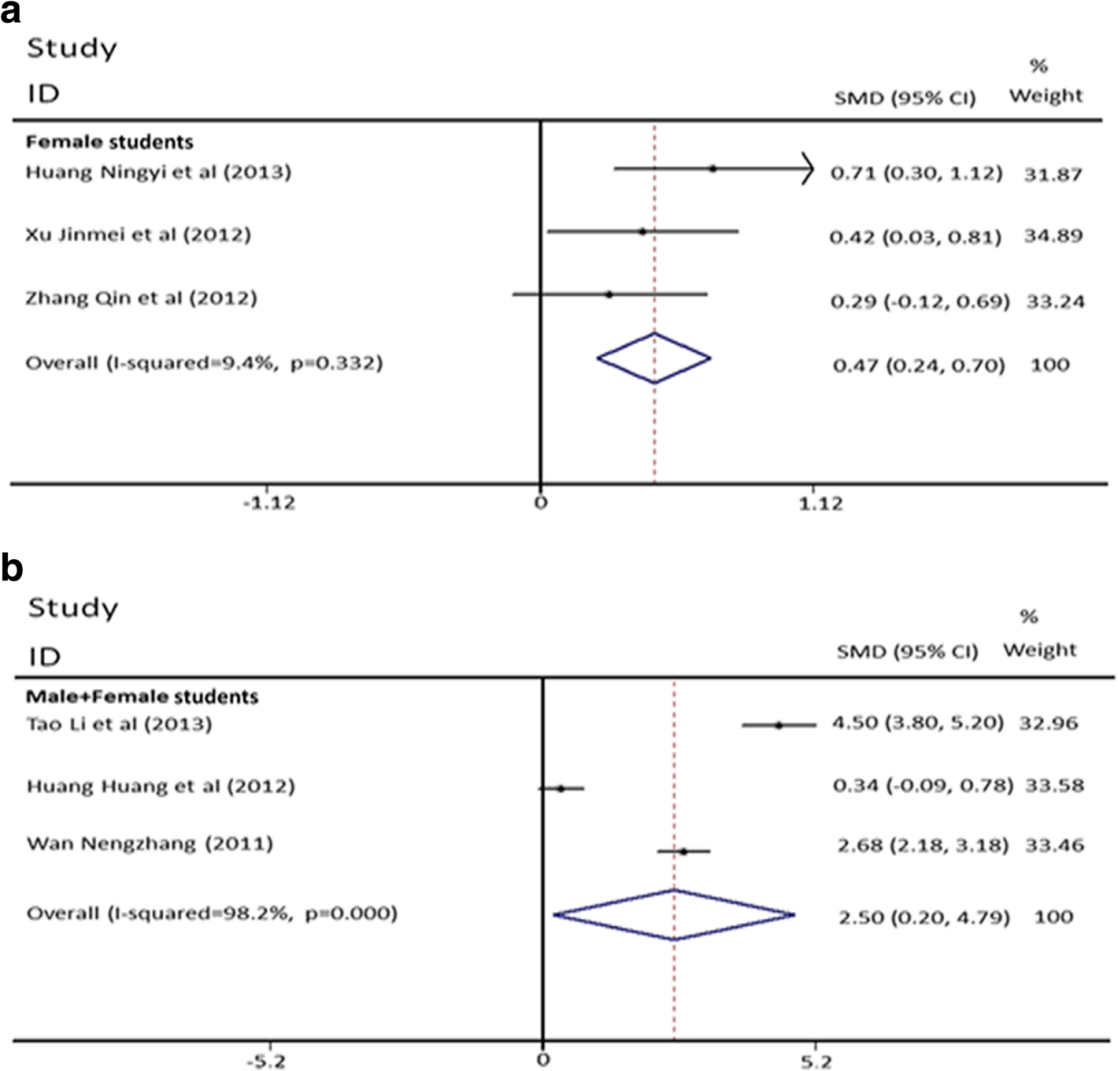
Forest plots for the effect of TBL on theoretical examination scores in medical college students grouped with gender (A: Female students. B: Male+Female students). The explanation for forest plot can be found in Fig. 2

We used meta-regression method to explore the sources of heterogeneity. Table 4 shows that the total methodological quality could not explain the source of heterogeneity (*P* = 0.975). We found the heterogeneity could be partially explained by the randomization (*P =* 0.021) (Table 4), which was supported by the decreased heterogeneity in the randomized designed group (for randomized designed group: *I*^2^ = 93.9%, *P*_heterogeneity_ < 0.001; for nonrandomized designed group: *I*^2^ = 98.1%, *P*_heterogeneity_ < 0.001). Meta-regression analysis further showed that education classification might be a contributing factor of heterogeneity in nonrandomized group (*P =* 0.073) (Table 5). Figure 3 shows that the heterogeneity was dramatically decreased in nonrandomized studies of undergraduate students (5-year program) (*I*^2^ = 57.5%, *P*_heterogeneity_ = 0.052, Table 5). In addition, in the pooled analysis of this group, we found TBL significantly increased examination scores in random-effects model (SMD = 4.39, 95% CI: 3.92–4.87, *I*^2^ = 57.5%, *P*_heterogeneity_ = 0.052) (Fig. 3) (Table 5). Meta-regression was next used in the medical college students (3-year program) to find the source of heterogeneity, which identified gender as a potential source of heterogeneity (*P* = 0.059) (Table 5). There was no heterogeneity in the pooled analysis of the medical college students of only females (*I*^2^ = 9.4%, *P*_heterogeneity_ = 0.332, Fig. 4, Table 5), in which we also found TBL significantly increased female students’ examination scores in fixed-effects model (SMD = 0.47, 95% CI: 0.24–0.70, *I*^2^ = 9.4%, *P*_heterogeneity_ = 0.332).

**Table 4:** Meta-regression analysis of 13 studies for exploration of the sources of heterogeneity

Factors	Coefficient	Standard error	95% Confidence interval	*P*
Quality score	−0.02	0.50	−1.12-1.09	0.975
Student number	0.38	0.60	−1.15-1.91	0.550
Randomization	−4.28	1.30	−7.61--0.95	0.021
Source of participants	2.69	0.98	0.16–5.21	0.041
Disciplines or curricula	1.08	1.31	−2.29-4.45	0.447
Gender	0.64	1.15	−2.31-3.58	0.602
Allocation concealment	1.18	1.85	−3.56-5.92	0.551
Control for important factors	0.39	1.38	−3.16-3.93	0.791

Meta-regression analysis, given the multi-collinearity, the meta-regression models were built for quality scores and methodological quality factors, respectively; randomization was coded as no(0) or yes(1); source of participants was coded as medical college students(0) or undergraduate students(1)

**Table 5:** Summary results of subgroup

	No. of studies	SMD (95%CI)	*I* ^2^	Heterogeneity *P* value	Meta regression *P* value
Total	13	2.46 (1.53–3.40)	98.00%	< 0.001	
Subgroup					
Education (No randomization)	11				0.073
Undergraduate students	5	4.39 (3.92–4.87)	57.50%	0.052	
Medical college students	6	1.47 (0.38–2.55)	97.10%	< 0.001	
Gender (Medical college students)	6				0.059
Female	3	0.47 (0.24–0.70)	9.40%	0.332	
Male+Female	3	2.50 (0.20–4.79)	98.20%	< 0.001	

*SMD* standardized mean difference, *CI* confidence interval;

*SMD (95%CI)* random-effects model was used when *P* value for heterogeneity test ≤0.10 (Total, Undergraduate students, Medical college students, Male+Female); otherwise, fix-effects model was used (Female);

Meta regression *P* value, represents the test for the significance of the effect modification across strata

### Sensitivity analyses

**Fig. 5:**
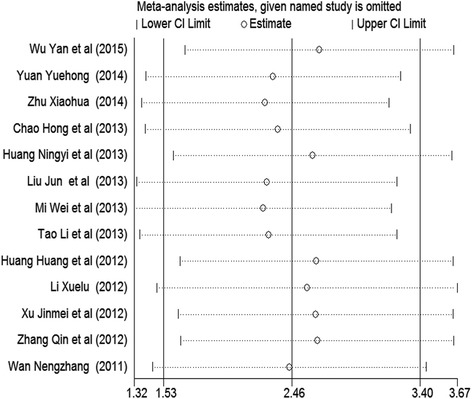
Sensitivity analysis assessing the influence of individual studies on the pooled analysis regarding the effect of TBL on students’ theoretical examination scores

When any research was removed from the model, the significant results of TBL effect on the students’ examination scores were unchanged in these models (SMD: 2.26–2.67, 95% CI: 1.32–3.67, *n* = 13) (Fig. 5). Consistent results of TBL effect on the students’ examination scores in fixed-effects model (SMD = 1.63, 95% CI: 1.51–1.76, *I*^2^ = 98.0%, *P*_heterogeneity_ < 0.001) were also observed (Additional file 1: Figure S1). All the above results indicated that the findings were robust.

## Discussion

Reports reveals that learning is more effective when students are actively involved in TBL, and TBL results in high student satisfaction in the USA [33–35]. TBL as an active learning pedagogical approach fits for the status of Chinese medical education, such as permission of a large student-teacher ratio to solve the problem of lack of teachers and classrooms [13]. However, TBL is still in its infancy in medical school in China, and its effectiveness still needs to be scientifically verified. This study is the first to evaluate the effectiveness of TBL on medical education in China by meta-analysis. We found that TBL improved student theoretical examination scores, learning attitude and learning skill compared to LBL pedagogy in the pooled analysis, and the positive effect of TBL on theoretical examination scores was also verified in sub-grouping analysis and sensitivity analysis, indicating the importance of the implement of TBL in China. Notably, these findings about TBL and traditional LBL were also supported by studies conducted in Japan and the USA [36–38], indicating the positive effectiveness of TBL in the present study may be not country-specific. As the topic about TBL and LBL continues to be studied globally, a detailed comparison of the effectiveness of TBL in medical education among different countries is required to be conducted in the future.

In China, undergraduate course (5-year program) focuses on theoretical and practical teaching, and its students often have better learning basis, while medical college course (3-year program) focuses on practical teaching. The students’ theoretical examination scores appeared positively related to education levels (meta-regression for all studies, Coefficient = 2.69, 95% CI:0.16–5.21, *P* = 0.041) (Fig. 3) (Tables 4-5), indicating the learning basis and learning objective may impact the effect of TBL on medical education. In addition, in the nonrandomized studies of undergraduate students with lower heterogeneity (*I*^2^ = 57.5%, *P*_heterogeneity_ = 0.052), significant effect was observed in the pooled analysis (Fig. 3), providing the evidence about the positive effect of TBL on medical education in undergraduate students in China.

We found in the medical college students of only females, the homogeneity was reached (*I*^2^ = 9.4%, *P*_heterogeneity_ = 0.332). The significant improvement of examination scores of TBL was observed when compared with LBL in the pooled analysis, verifying the positive effect of TBL on medical education in the medical college students in China.

### Limitations and future studies

However, limitations need to be addressed in our meta-analysis. There were only two randomized designed studies included in the present study [19, 28], and the sample size of one study was less than 100 [19]. In China, most educational studies were conducted based on the comparison of different classes for convenience. However, we should notice that this nonrandomized design may compromise the findings. In this meta-analysis, we observed that the randomization appeared to change the study results (meta-regression for all studies, Coefficient = − 4.28, 95% CI:-7.61--0.95, *P* = 0.021), emphasizing the importance of improving the educational study quality by using randomized study design. To better study the effect of TBL on medical education, well-designed and strictly controlled studies with established performing criterion including randomized controlled trials (RCTs) are still required.

Another limitation of this study is that, for the feasibility of meta-analysis due to the limited published studies, we didn’t include the comparison between TBL and other pedagogical approaches in this study. However, LBL is still the most commonly used pedagogy in theoretical teaching of medical education in China. Therefore, the effectiveness of any new pedagogical approach should be compared with LBL as a reference, which has a great practical significance. With the development of other new pedagogical approaches in the medical education in China, a more comprehensive comparison of different pedagogical approaches to test the effectiveness of TBL is required in the future.

The third limitation of current study is that, although this meta-analysis covered the major and overall results of teaching effectiveness in current published studies, the study was still limited in theoretical examination in those studies which might test memorization rather than measuring higher cognitive levels as well as the objectivity of measures of learning attitude and skill by student survey. Additionally, some other aspects of medical education are required to be investigated in the future. The included articles did not adopt standardized questionnaire survey, making the pooled analysis could not be conducted in some detailed aspects of teaching effect such as the effect on team spirit and oral expression. Therefore, more studies on this topic including both theory course and laboratory course are required which are performed with standardized study design and outcome assessment.

## Conclusions

In conclusion, the meta-analysis shows that TBL in theoretical teaching of medical education is more effective than LBL in improving learning knowledge, attitude and skills in China, providing evidence for the implement of TBL in medical education in China. TBL should be further gradually introduced into medical teaching programs. A future meta-analysis needs to be conducted to determine if the results from this meta-analysis continue to hold true with larger sample sizes. Medical schools should implement TBL with the consideration on the practical teaching situations such as students’ learning basis and objective which have been identified in this study. Furthermore, to improve the teaching quality in medical education in China, the effective way of integrating LBL and TBL as well as other pedagogical approaches needs to be further explored.

## Additional file

Additional file 1: **Figure S1.** Forest plot for the effect of TBL on examination scores compared with LBL (fixed-effects model). (DOC 2145 kb)

## Abbreviations

CBM: Chinese biomedical literature database
CNKI: Chinese national knowledge infrastructure
LBL: Lecture-based learning
SMD: Standardized mean difference
TBL: Team-based learning
VIP: Chinese scientific journal database
