# Supplementary material for: Meta-analysis on the effectiveness of team-based learning on medical education in China
Source: BMC Med Educ. 2018 Apr 10;18:77. doi: 10.1186/s12909-018-1179-1 (PMC5894173; doi:10.1186/s12909-018-1179-1)
Supplement: Supplementary file 1 — Figure S1. Forest plot for the effect of TBL on examination scores compared with LBL (fixed-effects model). (DOC 2145 kb) [file 12909_2018_1179_MOESM1_ESM.doc]

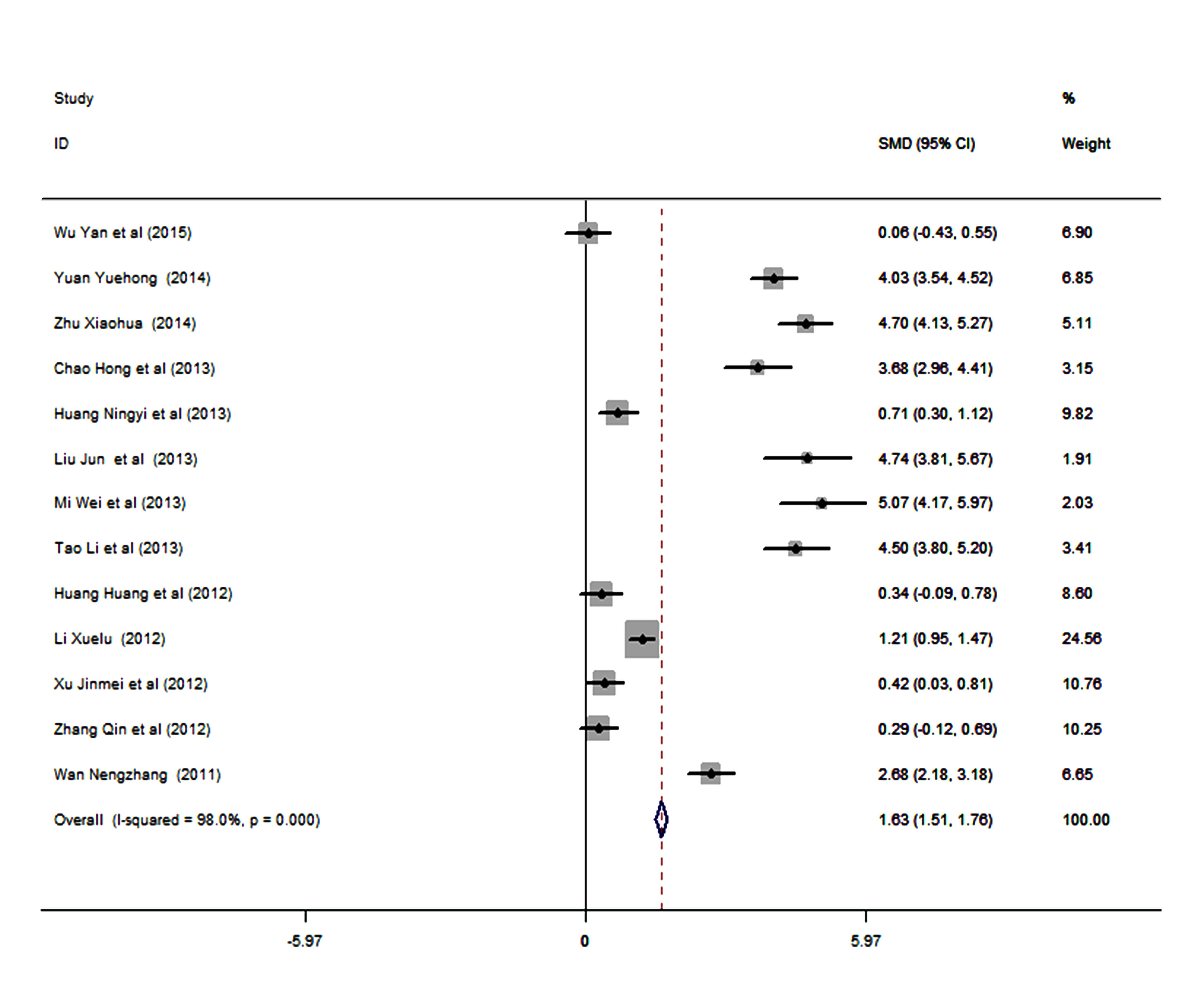


**Fig. S1 Forest plot for the effect of TBL on examination scores compared with LBL (fixed-effects model).**

Studies are plotted according to the last name of the first author and followed by the publication year in parentheses. Horizontal lines represent 95% CI. Each square represents the SMD point estimate of the study, and its size is proportional to the weight of the study. The diamond (and broken line) represents the overall summary estimate, with confidence interval given by its width. The unbroken vertical line is at the null value (SMD=0). CI, confidence interval; SMD, standardized mean difference.
